# A Novel “Slit Side View” Method to Evaluate Fluid Dynamics during Phacoemulsification

**DOI:** 10.1155/2018/5027238

**Published:** 2018-09-27

**Authors:** Hisaharu Suzuki, Tsutomu Igarashi, Toshihiko Shiwa, Hiroshi Takahashi

**Affiliations:** ^1^Department of Ophthalmology, Nippon Medical School Musashikosugi Hospital, 1-396 Kosugi-cho, Nakahara-ku, Kawasaki City, Kanagawa 211-8533, Japan; ^2^Department of Ophthalmology, Nippon Medical School, 1-1-5 Sendagi, Bunkyo-ku, Tokyo 113-8603, Japan

## Abstract

Due to recent technical advances in cataract surgeries, there has been a significant improvement in the safety and surgical outcomes of phacoemulsification. However, the corneal endothelium can be damaged during phacoemulsification by multiple factors. Therefore, we used a slit lamp to analyze the fluid dynamics of ophthalmic viscosurgical devices (OVDs) in the anterior chamber during phacoemulsification. In this experimental study, extracted porcine eyes were injected with OVDs stained with fluorescein through a side port of the eye and then fixed on a slit lamp microscope. After inserting a phaco tip, phacoemulsification simulation was then performed on the iris plane. Subsequent movements of OVDs in the anterior chamber were observed during the procedure by using a slit lamp microscope. Aspiration and removal of cohesive OVDs from the inside of the anterior chamber occurred within a few seconds after the ultrasonic vibration. Aspiration of dispersive OVDs occurred gradually, with some of the OVDs remaining on the side of the anterior chamber side in an irregular shape. This shape enabled the OVD to trap the air, thereby preventing the air from directly touching the corneal endothelium. Viscoadaptive OVDs remained inside the anterior chamber as a lump, with the infusion solution flowing between the corneal endothelium and the OVD, thus leading to the eventual aspiration of the OVD. Viscous dispersive OVDs remained as a lump between the corneal endothelium and the phaco tip. However, once the infusion solution flowed between the cornea and the OVD, the OVD detached from the corneal endothelium, indicating that this type would likely be aspirated and removed. This method, termed the “slit side view,” enables viewing of the movement of OVDs during surgery, as well as observation of the fluid dynamics in the anterior chamber.

## 1. Introduction

Today, most cataract surgeries are performed by using the phacoemulsification technique. Due to recent technical advances, there has been a significant improvement in the safety and surgical outcomes of phacoemulsification. However, the corneal endothelium can be damaged during phacoemulsification by multiple factors, such as excessive duration of the phacoemulsification [1–4]. Since the introduction of Healon® (sodium hyaluronate 1.0%) in 1980, many ophthalmic viscosurgical devices (OVDs) have become available. Moreover, OVDs play an important role in endothelium protection [5, 6]. Therefore, it is important to understand the dynamics of OVDs in the anterior chamber during phacoemulsification.

The surgical microscope is an essential apparatus when performing phacoemulsification. However, when using this microscope, observations are primarily made from the front, which complicates a clinician's ability to understand the detailed positional relationships that are present inside the anterior chamber. In contrast, a slit lamp microscope is used in outpatient clinics. When using this device, the anterior chamber space can be observed in detail. Therefore, we developed an observation method that uses a slit lamp microscope to obtain a detailed understanding of the movements that occur inside the anterior chamber during surgery. The purpose of this study was to evaluate the effectiveness of using a slit lamp to observe fluid dynamics in the anterior chamber during phacoemulsification.

## 2. Materials and Methods

This study was conducted in accordance with the ARVO Statement for the Use of Animals in Ophthalmic and Vision Research. The ethics committee ruled that approval was not required for the study. Extracted porcine eyes obtained from a local abattoir were used for the experiment.

Fluorescein (Ayumi Pharmaceutical) was applied to each of the OVDs to facilitate visualization. For each of the OVD staining methods, a 0.7 mg fluorescein examination test paper was divided into five equal parts, with the test paper then inserted into the tip of the syringe for each of the OVDs. OVD syringes were then stored vertically for 2 days in the refrigerator, which is our novel method to enable diffusion of the fluorescein into the OVDs. All OVDs used in the study were stored and used at room temperature.

**Figure 1:**
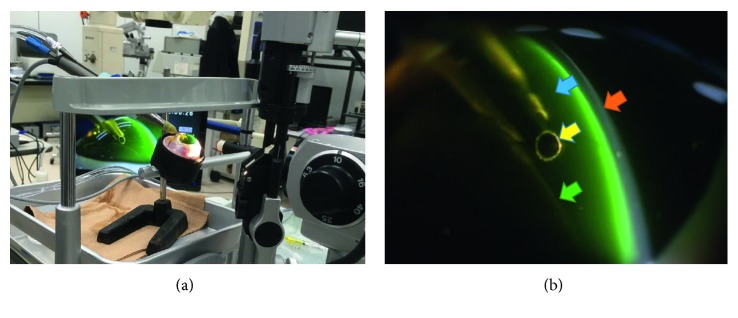
Laboratory arrangement used in the mechanical and porcine eye experiments: (a) overall picture. (b) Anatomical position in the anterior chamber: cornea (red arrow), OVD stained by fluorescein (blue arrow), US tip (yellow arrow), anterior capsule of the lens (green arrow).

By using an operation microscope, we first injected an OVD stained with fluorescein via the side port. The volume of each OVD was 0.4 ml. Second, we created a 2.4 mm incised corneal wound, making a continuous curvilinear capsulorhexis. Third, we transferred the porcine eye to the eyeball-fixing stand that was attached to the slit lamp microscope, inserted a phaco tip, and then fixed it over the iris surface. We observed the movement and behavior of the OVD inside the anterior chamber by using the slit lamp microscope 700GL (Takagi, Nagano-ken, Japan) (Figure 1(a)). We named this procedure the “slit side view” (SSV) (Figure 1(b)).

Phacoemulsification was performed in porcine eyes with the WhiteStar Signature PRO® System (Abbott Medical Optics, Santa Ana, CA, USA). The scale of light volume in the slit lamp microscope 700GL was set to 8/20. Ultrasound (US) oscillation was applied for 90 seconds. Observation points were 3, 25, 30, and 90 seconds. Phacoemulsification was performed with 20% power of longitudinal vibration using a 30-degree Signature Laminar® 20-gauge US tip. This method was used to perform several different intraoperative evaluations. We examined several OVDs during these evaluations, with differences in movement classified according to the type of OVD used.

We examined the movements for each of the OVDs inside the anterior chamber according to its type. These OVDs included the cohesive type (Opegan Hi®; Santen, Osaka, Japan), dispersive type [Shellgan® (Santen)], viscoadaptive type [Healon5® (Abbott Medical Optics)], and viscous dispersive type [DisCoVisc® (Alcon, Fort Worth, TX, USA)] (Table 1). We also examined a soft-shell technique [7] that uses a combined cohesive (injection volume: 0.3 ml) and dispersive (0.1 ml) type of OVD. In the soft-shell technique, we used undyed cohesive OVD. After injecting the dispersive OVD, the cohesive OVD was injected deliberately under the first OVD to push it forward against the corneal endothelium. The volume of the dispersive OVD was determined as 0.1 ml because it apparently seemed enough to coat the whole area of the corneal endothelium. The second OVD was injected until the anterior chamber was filled, and the leak of the OVD was confirmed. Moreover, in this group, we recorded the dynamics of OVD during injection with the slit side view. Recording parameters included a US power output of 20%, vacuum pressure of 200 mmHg, and bottle height of 75 cm. A Signature PRO® venturi pump was used for this part of the study. In all experiments, a 20G phaco tip was used. Three porcine eyes were evaluated for each OVD group.

## 3. Results

**Figure 2:**
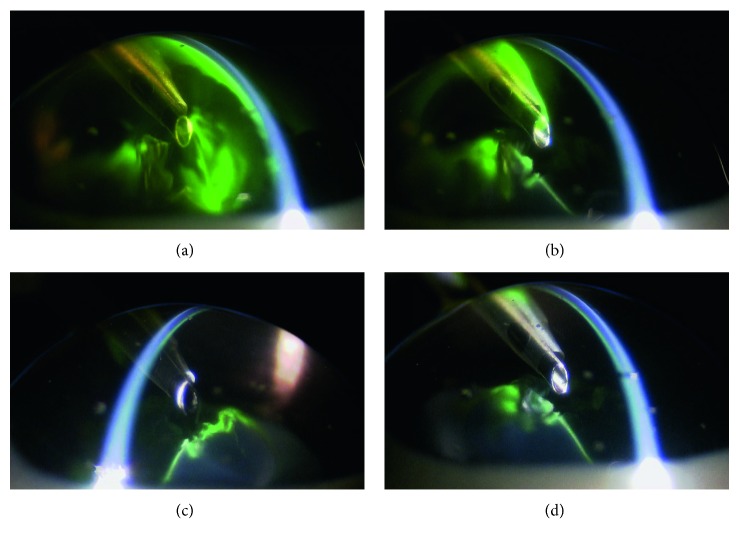
Cohesive type of ophthalmic viscosurgical devices (OVDs): (a) 3 seconds: the moment immediately after depressing the pedal of the pump. (b) 25 seconds: the OVDs are no longer washed out of the anterior chamber. (c) 30 seconds: cross-sectional view of the tip. (d) 90 seconds: the OVDs do not remain on the side of corneal endothelium.

**Figure 3:**
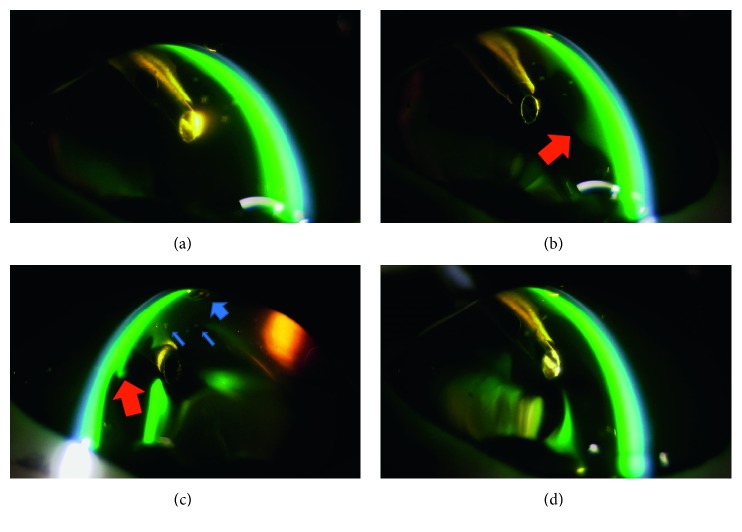
Dispersive type of ophthalmic viscosurgical devices (OVDs): (a) 3 seconds: OVDs remain fully on the corneal endothelium. (b) 25 seconds: the OVDs remain on the side of the anterior chamber side in an irregular shape, as if the OVDs had dripped down the side (arrow). (c) 30 seconds: cross-sectional view of the tip (red arrow indicates OVDs). Air is trapped in the OVD (blue arrow indicates airs). (d) 90 seconds: the OVDs remain on the side of the corneal endothelium.

**Figure 4:**
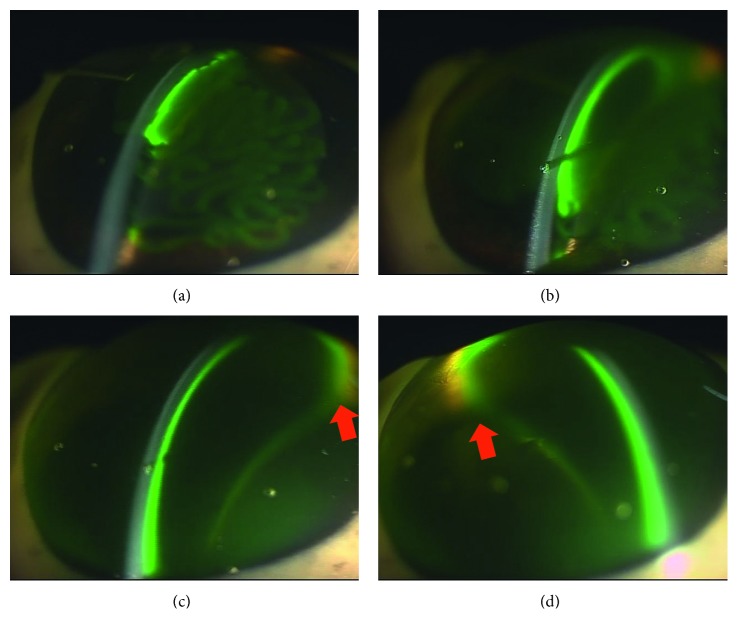
Creation of the soft-shell technique: (a) immediately after injecting the stained dispersive type of ophthalmic viscosurgical devices (OVDs). (b) The clear cohesive types of OVDs were then injected. (c) The dispersive types of OVDs were able to further spread on the corneal endothelial surface and on the anterior surface of the crystalline lens and the iris surface (arrow). (d) Cross-sectional view (arrow indicates OVDs).

**Figure 5:**
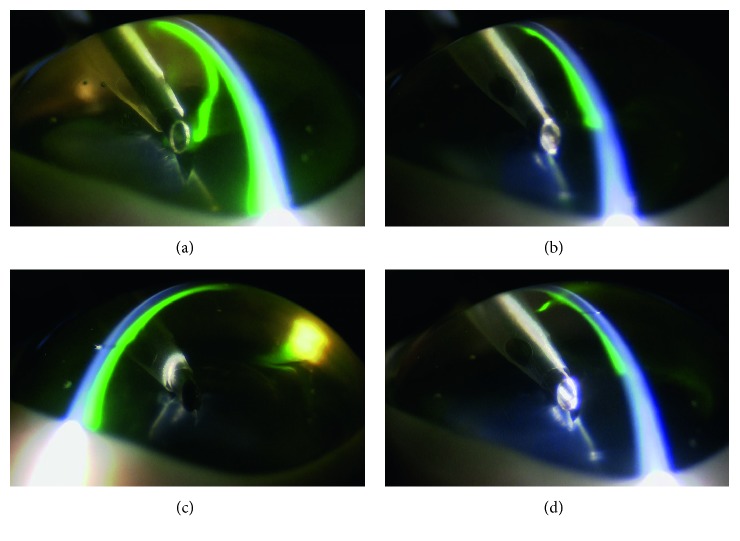
Soft-shell technique: (a) 3 seconds: after the application of ultrasound, the cohesive types of ophthalmic viscosurgical devices (OVDs) were immediately aspirated. (b) 25 seconds: the dispersive types of OVDs were retained on the surface of the corneal endothelium. (c) 30 seconds: cross-sectional view of the tip. (d) 90 seconds: dispersive types of OVDs formed a layer with a certain thickness on the surface of the corneal endothelium.

The imaging of the anterior chamber during phacoemulsification was performed at 3, 25, 30, and 90 seconds. Aspiration and removal of the cohesive type of OVDs from the inside of the anterior chamber occurred within a few seconds after the creation of the ultrasonic vibration (Figure 2). Aspiration of the dispersive type of OVDs occurred gradually, with some of the OVD remaining on the side of the anterior chamber side in an irregular shape, as if the OVD had dripped down the side. This shape enabled the OVD to trap the air and prevent the air from directly touching the corneal endothelium (Figure 3). The soft-shell technique uses a combination of the dispersive and cohesive types of OVDs. During the first step of this procedure, a dispersive type of OVD was injected, followed by a cohesive type. Results clearly showed that after being pressed by the cohesive type of OVD, the dispersive type of OVD was able to further spread on the corneal endothelial surface, the anterior surface of the crystalline lens, and the iris surface (Figure 4). After the application of US, the cohesive types of OVDs were immediately aspirated. However, the dispersive types of OVDs were retained on the surface for an extended period because they were able to form a layer of various thicknesses on the surface of the corneal endothelium (Figure 5).

**Figure 6:**
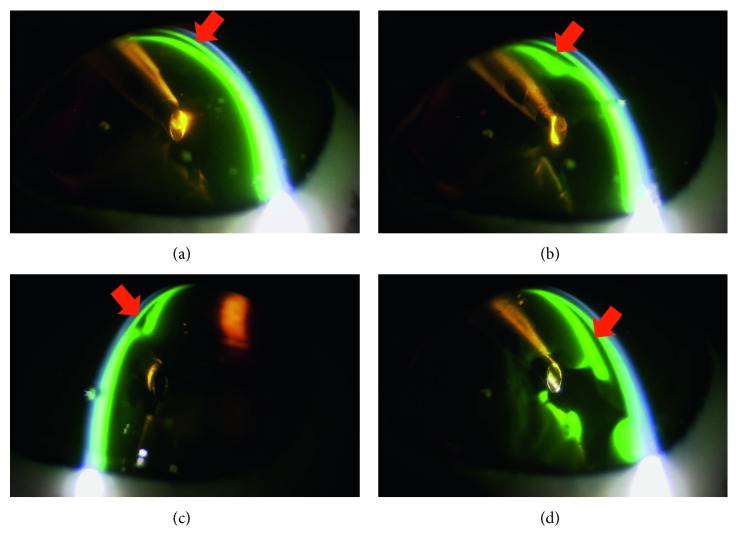
Viscoadaptive type of ophthalmic viscosurgical devices (OVDs): (a) 3 seconds: there is a clear gap between the corneal endothelium and the OVDs (arrow). (b) 25 seconds: the gap remains (arrow). (c) 30 seconds: cross-sectional view of the tip (arrow indicates gap). (d) 90 seconds: the range of the gap has expanded (arrow).

The next OVD studied was the viscoadaptive type. This type of OVD remained inside the anterior chamber as a lump, with the infusion solution flowing between the corneal endothelium and the OVD (Figure 6). When this occurred, the infusion solution often flowed between the corneal endothelium and the OVD from around the incised wound, thus leading to the eventual aspiration of the OVD. Typically, this type of finding cannot be readily be detected from the front by using a microscope. Furthermore, these findings suggest that, although the viscoadaptive type of OVD appeared to be effective against physical invasion of the nucleus, it may not be able to prevent microscopic invasions, such as by free radicals and cavitation.

**Figure 7:**
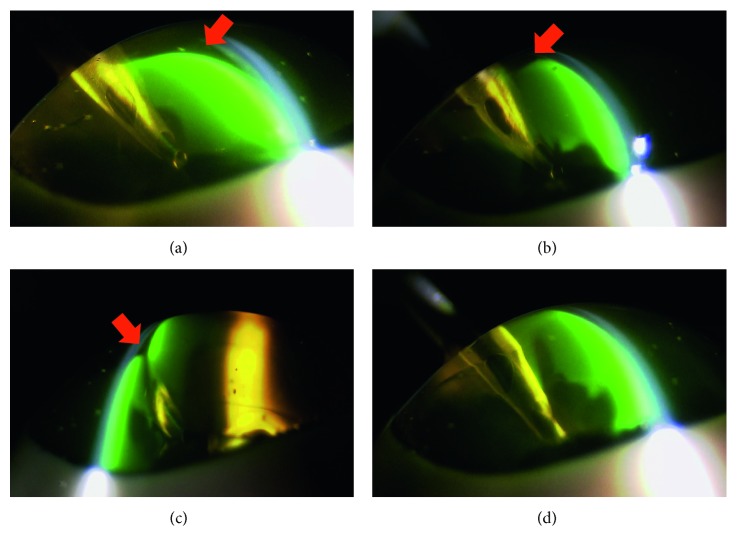
Viscous dispersive type of ophthalmic viscosurgical devices (OVDs): (a) 3 seconds: there is a clear gap between the corneal endothelium and the OVDs (arrow). (b) 25 seconds: the gap remains (arrow). (c) 30 seconds: cross-sectional view of the tip (arrow indicates gap). (d) 90 seconds: The perfusion has pressed the OVDs, and the gap is closed.

Subsequently, the viscous dispersive type of OVD remained as a lump between the corneal endothelium and the phaco tip (Figure 7). However, once the infusion solution flowed between the cornea and the OVD, the OVD detached from the corneal endothelium, thereby indicating that it is likely that this type would be aspirated and removed. Due to the force of infusion flow, the OVD was subsequently pushed again, thereby causing the slit to close.

## 4. Discussion

A variety of OVDs are available for cataract and intraocular surgeries. As there are many products already on the market, the advantages and disadvantages of each of the OVDs have been previously described [8–12]. However, when dealing with difficult cases, such as small pupils, corneal endothelium reduction, and shallow anterior chambers, a detailed understanding of the characteristics of these OVDs is necessary. In the eye, however, OVDs are clear and invisible. To overcome this issue, the classic method of staining an OVD with fluorescein has been used to observe dynamic changes in clinical and experimental studies [13]. Bissen-Miyajima reported that the use of a side-view camera (Handycam, DCR-PC300K, Sony), in addition to a surgical microscope, helped to discriminate the three-dimensional movement of the stained OVDs [14]. While this method is very useful, it is difficult to observe the cross section between the corneal endothelium and the phaco tip in detail. Holmén and Lundgren have reported that the use of a surgical microscope for the anterior segment analysis system (EAS-1000, Nidek Co., Ltd.) enabled comparison of the anterior chamber depth maintenance and retention capacities of the commercially available OVDs, within a porcine cadaver eye model during simulated phacoemulsification [15]. Although results obtained by this method allow better understanding of the retention of OVDs in the anterior chamber with partial observation, it remains impossible to analyze fluid dynamics and retention of OVDs in the anterior chamber during cataract surgery. The use of our new SSV method enables better understanding of the properties and characteristics of the OVDs along with the flow of fluids; thus, these findings can be used in actual clinical situations.

In the first step of our study, we examined several OVDs to better understand their characteristics. After depressing the pedal of the phacoemulsification machine, the cohesive types of OVDs were immediately aspirated. While this result demonstrates that this type of OVD can only provide minimal corneal-endothelial protective effects, it also indicates that these OVDs can be easily removed after inserting the intraocular lens. Furthermore, these OVDs are advantageous in terms of preventing increased intraocular pressure and infections after surgery. Additionally, we discovered that the dispersive type of OVD could only be aspirated gradually, with some of the OVD remaining as an irregular shape on the side of the anterior chamber, appearing almost as if the OVD had dripped down the sides of the chamber. This sort of shape enables the OVD to trap the air (Figure 3(c)); thus, the OVD can prevent the air from directly touching the corneal endothelium. Therefore, we believe that this type of OVD could provide strong corneal endothelial protective effects. However, if the nucleus and air is trapped, then a drop in visibility may occur. For example, in clinical situations that use the dispersive type of OVD, trapped nucleus fragments that have adhered cannot be directly removed by the tip. However, they can be detached by infusion flow, so they can be processed under conditions that have a minimal influence on the corneal endothelium. Thus, using SSV to help determine and better understand the characteristics of the OVDs is beneficial for the overall clinical procedure.

Arshinoff reported that the soft-shell technique, which uses different types of viscoelastic substances, ranging from cohesive to dispersive, can be used to protect the corneal endothelium during phacoemulsification [7]. Furthermore, Arshinoff showed that the soft-shell technique can greatly facilitate cataract surgery by using the best properties of the higher-viscosity cohesive and lower-viscosity dispersive viscoelastic agents, while eliminating the disadvantages of both. By using our SSV methodology, we were able to confirm the advantages of this technique. When performing the soft-shell technique, a dispersive type of OVD is first injected, followed by a cohesive type. In our current experiment, pressure from the cohesive type of OVD caused the dispersive type of OVD to spread on the corneal endothelial surface, the anterior surface of the crystalline lens, and even further on the iris surface. After the application of US, the cohesive types of OVDs were immediately aspirated. However, since the dispersive types of OVDs had formed a layer of various thicknesses on the surface of the corneal endothelium, it was possible to retain these OVDs over a long period of time [5]. Comparing to the dispersive OVD alone, the soft-shell technique is thought to be better in the coating effect on the corneal endothelium because in the soft-shell technique, the cohesive OVD can push the dispersive OVD equally against the corneal endothelium as shown in Figures 3 and 5. These findings appear to confirm the results of the clinical studies of Miyata et al. [16], who reported that the soft-shell technique was superior regarding protection of the corneal endothelium. In both the viscoadaptive and viscous dispersive types of OVDs, the infusion solution flowed between the corneal endothelium and the OVD. Typically, this finding cannot be readily detected from the front by the use of a normal surgical microscope. As a result, if a problem with the irrigation occurs, this may cause corneal endothelium dysfunction. Therefore, regarding protection of the corneal endothelium, this finding suggests that it is important to know the direction of the irrigation during surgery. From that point of view, similar analysis on the effect of irrigation/aspiration without US oscillation should be done in the future study.

Furthermore, it is necessary to investigate whether the direction of the infusion solution can affect the residual OVD. However, since we confirmed that these two OVDs remained in the anterior chamber as a lump, it is possible that they might serve as physical obstacles and prevent collision with the nucleus [17].

## 5. Conclusions

The SSV method enables us to follow the movement of OVD during surgery and to observe changes in the anterior chamber in accordance with the machine settings. Therefore, this method has the potential to serve as an outstanding evaluation technique that can be used to help perform safe ocular surgery. In this experiment, the US tip is fixed, whereas in the clinical setting, the US tip is mobile. In the next experiment, it is necessary to study the dynamics of OVD in the anterior chamber when the US tip is moved.

## Figures and Tables

**Table 1:** The viscoelastic types.

Chemical names (brand names)	Viscosity	Composition
Cohesive (Opegan Hi®)	Intrinsic viscosity 25∼45 (dL/g)	1% sodium hyaluronate
Dispersive (Shellgan®)	35000∼60000 mPa s (25°C, shear rate 2/s)	3% sodium hyaluronate
		4% chondroitin sulfate sodium
Viscoadaptive (Healon5®)	Zero shear viscosity: about 7 million (mPa s)	23% sodium hyaluronate
Viscous dispersive (DisCoVisc®)	Viscosity (shear rate 1/s, 25°C): 75000 ± 35000 (mPa s)	1.65% sodium hyaluronate
		4% chondriotin sulfate sodium

Viscosity is the commercially published data of each OVDs.

## Data Availability

No data were used to support this study.
